# Quantitative trait loci controlling *Phytophthora cactorum* resistance in the cultivated octoploid strawberry (*Fragaria* *×* *ananassa*)

**DOI:** 10.1038/s41438-019-0136-4

**Published:** 2019-05-01

**Authors:** Charlotte F. Nellist, Robert J. Vickerstaff, Maria K. Sobczyk, César Marina-Montes, Fiona M. Wilson, David W. Simpson, Adam B. Whitehouse, Richard J. Harrison

**Affiliations:** Department of Genetics, Genomics and Breeding, NIAB EMR, New Road, East Malling, ME19 6BJ UK

**Keywords:** Agricultural genetics, Genome-wide association studies

## Abstract

The cultivated strawberry, *Fragaria × ananassa*  (*Fragaria* spp.) is the most economically important global soft fruit. *Phytophthora cactorum*, a water-borne oomycete causes economic losses in strawberry production globally. A bi-parental cross of octoploid cultivated strawberry segregating for resistance to *P*. *cactorum*, the causative agent of crown rot disease, was screened using artificial inoculation. Multiple putative resistance quantitative trait loci (QTL) were identified and mapped. Three major effect QTL (*FaRPc6C*, *FaRPc6D* and *FaRPc7D*) explained 37% of the variation observed. There were no epistatic interactions detected between the three major QTLs. Testing a subset of the mapping population progeny against a range of *P. cactorum* isolates revealed no significant interaction (*p* = 0.0593). However, some lines showed higher susceptibility than predicted, indicating that additional undetected factors may affect the expression of some quantitative resistance loci. Using historic crown rot disease score data from strawberry accessions, a preliminary genome-wide association study (GWAS) of 114 individuals revealed an additional locus associated with resistance to *P*. *cactorum*. Mining of the *Fragaria vesca* Hawaii 4 v1.1 genome revealed candidate resistance genes in the QTL regions.

## Genetics: Closing in on crown rot-resistant strawberries

Crown rot results in major economic losses for strawberry growers; now three regions of the strawberry genome have been identified which are associated with resistance to the disease. A better understanding of these genetic mechanisms may lead to the development of more crown rot-resistant plants. Richard Harrison at NIAB EMR in Kent, UK, and colleagues used quantitative trait loci mapping to pinpoint specific regions of the cultivated strawberry genome associated with resistance to *Phytophthora cactorum*, the water-borne pathogen that causes crown rot. These regions appear to influence disease susceptibility independently of one another, but together account for 37% of variance in resistance to *P. cactorum*. A further genome wide association study identified another locus associated with rot resistance. Further work is needed to elucidate the mechanism by which genes clustering in these regions affect disease susceptibility.

## Introduction

The cultivated strawberry, *Fragaria* *×* *ananassa* (*Fragaria* spp.) is the most economically important global soft fruit and is an integral part of the diet of millions of people^1^. Until recently, the major strategy for disease control in strawberry production relied heavily upon pre-plant fumigation and chemicals. The withdrawal of methyl bromide along with other active chemicals, including fungicides and soil fumigants are increasing the challenges in field strawberry production, resulting in a rise of occurrences and severities of some once well controlled diseases^2^. A switch to producing strawberries in soilless substrate is now common practice across the world. The soilless substrate system offers many advantages, including the benefit of separating the strawberries from the infected soil^3^. This has resulted in a reduction in the prevalence of some soil-borne diseases, but not for water-borne pathogens such as the hemibiotrophic oomycete, *Phytophthora cactorum*. *P. cactorum* (Lebert and Cohn) Schröeter is a destructive pathogen, that can infect a wide variety of plant species, causing serious damage in both ornamental and agricultural crops^4^. It is the causative agent of strawberry crown rot^5^ and strawberry leather rot^6^, affecting the fruit. Both diseases are reported to cause economic losses in strawberry production globally; in Norway in 1996/1997 there were reports of plants losses of up to 40% caused by crown rot^7^ and in 1981 reports of commercial farms in Ohio described crop losses from leather rot of 20–30%^8^. Amplified fragment length polymorphism (AFLP) analysis of *P. cactorum* isolates of crown rot and leather rot showed they are distinctly different from each other and from *P. cactorum* isolated from other hosts^9^. No correlation has been found between resistance to crown rot and resistance to leather rot^10^.

Strawberry plants infected with *Phytophthora* crown rot can often appear stunted; the youngest leaves are usually the first to wilt, followed by the older leaves, eventually resulting in the collapse and death of the plant^11^. Red-brown lesions and longitudinal splits can be observed within the crown^12^. Sexually produced oospores are the primary source of inoculum; these are the resting spores that can persist in the soil or infected plants for many years^11^. Under the conducive conditions of saturated soil, oospores germinate to produce sporangia which release the motile asexual life stage, zoospores. Zoospores are chemotactically attracted to nearby roots^13^, where they attach to the root surface, encyst and penetrate the root epidermis^14^. Once inside the host, intracellular growth leads to the development of haustoria, the site where the acquisition of nutrients for growth and sporulation occurs^14^.

The public breeding programme at NIAB EMR (East Malling, Kent, UK), since its establishment in 1983, has successfully released 43 strawberry cultivars to the Northern European market. Efforts have focused on combining excellent fruit quality with high yield, low percentage waste and resistance to filamentous diseases. Breeding for disease resistance is a high priority for many breeding programmes across the world. There has been extensive research investigating qualitative (major gene) resistance to *Phytophthora* species (for a selection of *R* gene—*Avr* gene interactions see Table 2 in Vleeshouwers et al.^15^), however, much less is known about quantitative resistance (multiple genes, each of partial effect) to *Phytophthora* species. Quantitative trait loci (QTL) mapping is a technique for pinpointing genes controlling complex polygenic traits to specific regions of the genome, through statistical analysis. Previous studies have identified resistance to *P. cactorum* in the octoploid strawberry and it appears to be under polygenic control^16–18^, with a major locus, *FaRPc2*, recently reported on linkage group 7D^19^. Variation in resistance has been also observed in the wild progenitors of *F*. × *ananassa*; *Fragaria chiloensis* and *Fragaria virginiana* populations^20^.

The cultivated strawberry (2*n* = 8× = 56) is an allo-polyploid outbreeder with a genome comprised of four comparable homeologous sets of diploid chromosomes^21,22^. The octoploid genome is estimated to be 698 Mb; 80% of the size of quadrupling the diploid genomes (~200 Mb each)^23^. The four sub-genomes are named A–D, based on the similarity of the sub-genome to *Fragaria* *vesca*^24^. The most similar sub-genome was named A, the second most similar was named B (similar to the wild diploid *Fragaria iinumae*), the third most similar was named C and the least similar was named D. This sub-genome denotation has been used in several other studies in the cultivated strawberry^19,25–27^.

The development of the 90K single-nucleotide polymorphism (SNP) Affymetrix^®^ IStraw90 Axiom^®^ Array^28^ has aided genetic studies and marker-assisted breeding. The genomes of 19 octoploid and 6 diploid strawberry accessions were sequenced to serve as resources for SNP discovery. Nine octoploids were used for SNP filtering, which included the current mapping parents ‘Emily’ and ‘Fenella’. A high percentage of SNP markers were designed sub-genome-specific to unravel the complexity of the allo-octoploid genome and allow accurate scoring. However, its widespread use was limited by cost. A smaller, cheaper version of the array, Axiom^®^ IStraw35 384HT, has been developed by combining mapped SNP probes from multiple groups from across the world and contains just over 34,000 markers^29,30^.

The genus *Phytophthora* comprises of numerous destructive crop pathogens^31^. The most extensively studied are *Phytophthora infestans* (late blight of potato and tomato) and *Phytophthora sojae* (root and stem rot of soybean). The majority of *R* genes identified in potato against *P. infestans* belong to the coiled-coil, nucleotide-binding, leucine-rich repeat (CC-NLR) class of intracellular immune receptors^32^. The corresponding avirulence (*Avr*) genes identified belong to the RxLR (arginine, any amino acid, leucine and arginine) class of effectors^32^. These secreted, modular effectors have an RxLR motif for translocation into the host cell with a quickly evolving effector domain at the C-terminus. A recent study on *P. cactorum* identified 199 RxLRs in a pathogenic strawberry isolate^33^. In comparison to the intracellular NLR resistance genes against RxLRs, fewer extracellular resistance genes have been characterised against *Phytophthora*. However, the few that have, have been associated with resistance to multiple *Phytophthora* pathogens. Cell surface l-type-lectin-RLKs (receptor-like kinases) have been associated with resistance to *Phytophthora brassicae*, *Phytophthora capsici* and *P. infestans*^34–36^. An extracellular receptor-like protein (RLP), elicitin response (ELR), was identified in a wild species of potato. ELR was found to recognise elicitin proteins from a diverse set of *Phytophthora* species, including *P. infestans*, *P. sojae* and *Phytophthora cryptogea*^37,38^.

In this study, the genetic basis of quantitative resistance to *P. cactorum* was investigated in a bi-parental cross of the cultivated octoploid strawberry (*F*. × *ananassa*). The mapping of resistance in controlled glasshouse experiments and the identification of QTL associated with resistance is reported. Furthermore, using historic crown rot disease score data, a preliminary genome-wide association study (GWAS) was conducted to investigate the presence of putative QTL within the wider germplasm. Subsequent to the identification of resistance QTL, the diploid strawberry reference genome (*F. vesca* Hawaii 4 v1.1) was mined for candidate resistance genes.

## Materials and methods

### Strawberry plant material

The mapping population used in this study was a cross between the cultivated June bearing strawberry cultivars ‘Emily’ × ‘Fenella’. ‘Emily’ is an early season variety with resistance to powdery mildew (*Podosphaera aphanis*), bred by NIAB EMR (formally HRI-East Malling) and released in 1995. It is moderately susceptible to *P. cactorum*. ‘Fenella’ is a mid-late season variety with good resistance to Verticillium wilt (*Verticillium dahliae*) and crown rot (*P. cactorum*), bred by NIAB EMR (formally East Malling Research) and released in 2009. The F_1_ full sib family of 181 individuals were clonally propagated by pinning down runners; the 181 progeny were planted in a field at the East Malling site (NIAB EMR) in May 2014 and grown under netting. The strawberry runners were pinned down in beds and grown on for six months, from July 2014 to January 2015. The clones were then dug up, the excess soil was shaken off and the bare-rooted plants were transferred into a 2 °C cold-store for one week, before being transferred to a −2 °C cold-store for at least two months. Plants were brought out of cold-storage and potted into 9 cm diameter pots (Soparco) and dead leaves were removed. Plants were grown in a glasshouse compartment maintained at 20 °C during the day and 15 °C at night on a 16/8 h, day/night light cycle, for three weeks before inoculation with *P. cactorum* isolates.

Strawberry plant material for the preliminary GWAS (see Table S1 for list of accessions) were also pinned down in fields and followed the same method as above over multiple years. Details of the years tested and isolates used can be found in Table S1.

### *Phytophthora cactorum* isolates

The main *P. cactorum* isolate used in this study for the bi-parental QTL mapping was P414. Isolates P404, P415 and P416 were used to screen the 15 representative progeny from three sensitivities (five lowly diseased individuals, five intermediate response individuals and five highly diseased individuals) of the bi-parental cross. The isolates used in the preliminary GWAS were P371, P372, P404, P407, P412, P413, P414 and P416. All isolates are known to be pathogenic to *F*. × *ananassa*, having been isolated from infected strawberry plants. Isolates of *P. cactorum* were maintained on V8-juice (Arnotts Biscuits Limited) agar (200 ml V8-juice, 8–9 ml 1M KoH (to adjust to pH 7.0; Sigma-Aldrich), 20 g Agar (Fisher BioReagents) and 800 mL distilled water, autoclaved) at 20 °C in the dark.

### *P. cactorum* zoospore production

Ten mm discs were cut from the margins of actively growing cultures of *P. cactorum* on V8-juice agar and placed into empty 90 mm triple-vented petri dishes (five per plate; Thermo Scientific). The plates were then carefully flooded with diluted compost extract (50 g compost in 2 L dH_2_O for 16 h in the dark at room temperature, then filtered through Whatman 113V Wet Strengthened 150 mm filter paper; and dH_2_O 1:1, stored in the fridge) and sealed with Parafilm (Bemis Company). Plates were placed in an incubator set at 20 °C with lights on continuously for 48 h to stimulate sporangia development. After 48 h, the diluted compost extract was poured off and replaced with fresh diluted compost extract. The plates were placed into a fridge (~4 °C) for 45 min and then moved onto the bench at room temperature (~20 °C) for 45 min. The inoculum suspension was then vacuum filtered and kept on ice. The concentration of zoospores was calculated using a haemocytometer and the concentration for artificial inoculation was adjusted to 1 × 10^4^ zoospores per mL^39^.

### Strawberry inoculation assays

The pathogenicity screens were carried out under controlled conditions in a glasshouse. Compartments were maintained at 20 °C during the day and 15 °C at night on a 16/8 h, day/night light cycle for four weeks after inoculation with *P. cactorum*. The ‘Emily × ‘Fenella’ progeny screens were performed in six experiments. The first two experiments comprised of one replicate mock inoculated and one replicate artificially inoculated with *P. cactorum* isolate P414. The other four experiments were comprised of two replicates artificially inoculated with *P. cactorum* isolate P414. The 15 representative progeny from three sensitivities (five lowly diseased individuals, five intermediate response individuals and five highly diseased individuals) of the bi-parental cross were screened with the three other *P. cactorum* isolates (P404, P415 and P416) in a separate experiment and inoculated separately. Plants for all screens were arranged in a randomised block design for each set of replicates. In total, ten replicates of each strawberry genotype were artificially inoculated with a suspension of *P. cactorum* zoospores. Fifteen mm wounds were made using a scalpel at the base of one petiole per plant (Figure S1) and the strawberry plants were sprayed with ~5 mL of 1 × 10^4^ zoospore suspension. For each strawberry genotype, two plants were mock inoculated by wounding in the same way and inoculated with ~5 mL diluted compost extract. To maintain humidity, plants were completely covered with clear plastic sheeting for 48 h. Plants were scored following a slightly modified version of Bell et al.’s disease scale^40^. Foliage was assessed visually for the presence of wilting symptoms once a week. The scores 8, 7, 6 and 5 were assigned if the plant died during the first, second, third or fourth week after inoculation, respectively. After four  weeks, the plants were cut open longitudinally and the crowns were assessed on a scale of 1–5; 1—healthy (0% infection), 2—up to 25% infection, 3—26–50% infection, 4—51–75% infection and 5—76–100% infection.

### Analysis of disease scores

The data for the ten replicates of each genotype was averaged and a mean crown rot disease score was used for further analysis. Statistical analyses were performed using R (v3.2.2, “Fire Safety”^41^). The raw data was tested for homogeneity of variance using Levene’s test. The mean crown rot disease data for the ‘Emily’ × ‘Fenella’ progeny was tested for normality using the Shapiro–Wilk normality test. Broad sense heritability (*H*^2^) was calculated, *H*^2^ = *V*_*G*_/*V*_*P*_, where V_*G*_ is the total genetic variance and *V*_*P*_ is the total phenotypic variance.

### DNA extraction and genotyping

Young emerging leaf samples were collected in 2 mL microcentrifuge tubes along with two ball bearings and flash frozen in liquid nitrogen. Frozen leaf samples were ground to a fine powder for 2 min at 60 o/m (oscillations per minute) using a TissueLyser (Qiagen). Genomic DNA (gDNA) was extracted using the DNeasy kit (Qiagen) following the manufacturer’s protocol and eluted in 60 μL Buffer AE. gDNA quantity and purity were determined using the NanoDrop (ND-1000, Thermo Scientific) spectrophotometer. In addition, the Qubit 2 Fluorometer (Thermo Fisher Scientific) was used to accurately measure the quantity. gDNA of ‘Emily’ and ‘Fenella’, the 181 progeny and 57 strawberry accessions, were sent to Oxford Genomics Centre for genotyping on the Affymetrix^®^ IStraw90 Axiom^®^ Array^28^. Later, a further 55 strawberry accessions were genotyped on the Affymetrix^®^ IStraw35 Axiom^®^ Array^30^, following the same sample preparation method.

### Linkage analysis of the bi-parental cross of ‘Emily’ × ‘Fenella’

Initial genotype calls were made using Affymetrix Power Tools (version 1.16.1) and the R package SNPolisher (version 1.5.0). Further filtering used custom Python scripts, part of the Crosslink package (https://github.com/eastmallingresearch/crosslink) to remove markers with strong segregation distortion^42^. A bi-parental genetic map of ‘Emily’ × ‘Fenella’ was produced using the 181 progeny and Crosslink software^42^. The same pipeline was also used to generate bi-parental maps from IStraw90 data from four additional crosses: ‘Redgauntlet’ ×  ‘Hapil’, ‘Flamenco’ × ‘Chandler’, ‘Capitola’ × ‘CF1116’ (INRA, France) and ‘Camarosa’ × ‘Dover’ (CRAG, Spain). Custom Python and R scripts were used to create a consensus genetic map from all five bi-parental maps, totalling 35,154 markers across the 28 linkage groups. Further custom scripts adjusted the fine scale marker ordering of the consensus map to match the *F. vesca* genome v2.0^22^, whilst identifying and correcting probable *F. vesca* genome assembly errors. The resulting hybrid consensus map was used to inform the ordering of the ‘Emily’ × ‘Fenella’ map, which now reports the physical position of each marker on a ‘pseudo-octoploid’ chromosome, rather than a centimorgan position on a genetic map. The four sub-genomes of *F*. × *ananassa* were assigned the letters A–D in the ‘Emily’ × ‘Fenella’ linkage map, in the same order as described by van Dijk et al.^24^ (confirmed by comparison with the unpublished ‘Holiday’ × ‘Korona’ SNP map, based on the published simple sequence repeat, SSR, map, E. van de Weg, personal communication) and used in several other studies^19,25–27^.

### QTL identification

Histograms of mean crown rot disease scores were visualised and tested for normality (QQ-plot). The raw mean data (Shapiro–Wilk test, *W* = 0.96877, *p* < 0.0004) were used for QTL analysis. QTL identification was performed using Kruskal–Wallis (K–W) non-parametric ANOVA using the physical positions of SNPs found in ‘Emily’ and ‘Fenella’, extracted from a consensus map. Identification of QTL specific to one parent and QTL that are present in both parental genotypes were estimated with the K–W method, eliminating the need to perform separate QTL analysis on the two parental maps, as performed in a similar analysis by Cockerton et al.^43^. K–W analysis identifies markers linked to single traits/QTL individually and produces a *K* statistic. Putative associated regions with resistance to *P. cactorum* were identified if *p* < 0.05, and the most significant (focal) marker was selected.

A stepwise linear regression model was performed to estimate the effect of each putative associated region. The most non-significant putative associated region was removed from the model one at a time until only significant putative associated regions remained (*p* > 0.05). The input order of putative associated regions in the model did not influence the estimate of effect. The combination of the putative associated region effect sizes were used to calculate predicted means for each individual. The predicted means were plotted against observed average scores and their coefficient of determination (*r*^2^) was calculated.

A further stringent test was applied to narrow down the number of putative QTL for breeders to focus on. The Bonferroni correction was applied to the K–W *p* values to control the false-positive (type I error) rate. It was calculated using: critical *p* value (*α*)/number of comparisons being made. Only QTL with *p* values more significant than the Bonferroni correction were investigated further. Homogeneity of variance of the significant QTL was determined using Levene’s test. Analysis of variance (ANOVA) was performed to test for epistatic interactions between the major effect QTL that passed the Bonferroni correction.

To investigate if the major QTL were masking other QTL, multiple-QTL models (MQM) mapping was performed in MapQTL^®^ 5^44^. Using the IStraw35 markers only, the most significant marker for each of the three major QTL (Affx-88882258, Affx-88880166 and Affx-88902178) were selected as co-factors in an approximate multiple QTL analysis, restricted MQM mapping (rMQM).

### Testing for progeny–isolate interactions

ANOVA was performed to test for interactions between the progeny from the three sensitivity groups of the bi-parental cross and the four *P. cactorum* isolates; P414, P404, P415 and P416.

### Preliminary genome-wide association study

A preliminary GWAS was performed using historic crown rot score data of 114 strawberry accessions (detailed in Table S1), collected between 1995 and 2017, using a mixture of two *P. cactorum* isolates each year: pre-2006—isolates P371 and P372, 2006–2009—isolates P412 and P413, 2010–2011—isolates P412 and P407 and 2012–2017—isolates P404 and P416. Along with these, some individuals were tested with a single isolate, P414 in 2016.

SNPs showing at least 5% minor allele frequency were assessed for association with resistance to crown rot in the 114 strawberry accessions using both PLINK^45^ and TASSEL (Trait Analysis by Association, Evolution and Linkage)^46^ (*p* < 0.00005; https://github.com/harrisonlab/popgen/blob/master/snp/gwas_quantitative_pipeline.md). Population structure was taken into account by clustering individuals into homogeneous subsets, based upon pair-wise identity by descent distance. This enabled the visualisation of substructure through multidimensional scaling and the production of quantitative indices of population genetic variation, which was used as covariates in the following association analysis. The Benjamini–Hochberg procedure was applied to reduce the false-discovery rate and corrected *p* values that were lower than *p* = 0.05 were considered to be potential QTL.

### Mining of candidate resistance genes in *F. vesca*

The most significant SNP markers for each QTL were plotted on the *F. vesca* Hawaii 4 v1.1 genome^47^ in Geneious (v10.1.2), along with generic feature format files with the positions of NLR, RLK and RLP gene models. The number of genes in each of these classes within 1 Mbp either side of the most significant marker for each QTL were determined.

## Results

### Variation observed in resistance to *P. cactorum* in the bi-parental cross

Crown rot disease severity was found to vary in a genotype-dependent manner. In the most susceptible individuals, total plant collapse occurred one or two weeks after inoculation and 100% necrosis of the crown was observed (crown rot disease scores 7/8, respectively). The mock-inoculated plants remained disease-free. The distribution of crown rot disease severity for the 181 individuals of the ‘Emily’ and ‘Fenella’ mapping population had a unimodal distribution pattern, with a slight skew towards resistance (Figure S2). The means of the raw data were normally distributed and were used for QTL mapping (Figure S2). The broad sense heritability was calculated to be *H*^2^ = 0.58.

### Whole-genome linkage map assembled

A whole-genome linkage map comprising of 11,598 SNP markers was assembled using the IStraw90 markers of the ‘Emily’ × ‘Fenella’ progeny (File S1) and the programme Crosslink^42^. The map was resolved into 28 linkage groups, representing the four sub-genomes of each, of the seven chromosomes of *F*. × *ananassa*. Using only the IStraw35 markers (a subset of IStraw90 markers) to produce the map, 8,348 SNP markers were assembled into 28 linkage groups (File S2).

### Significant putative resistance QTL identified

**Fig. 1:**
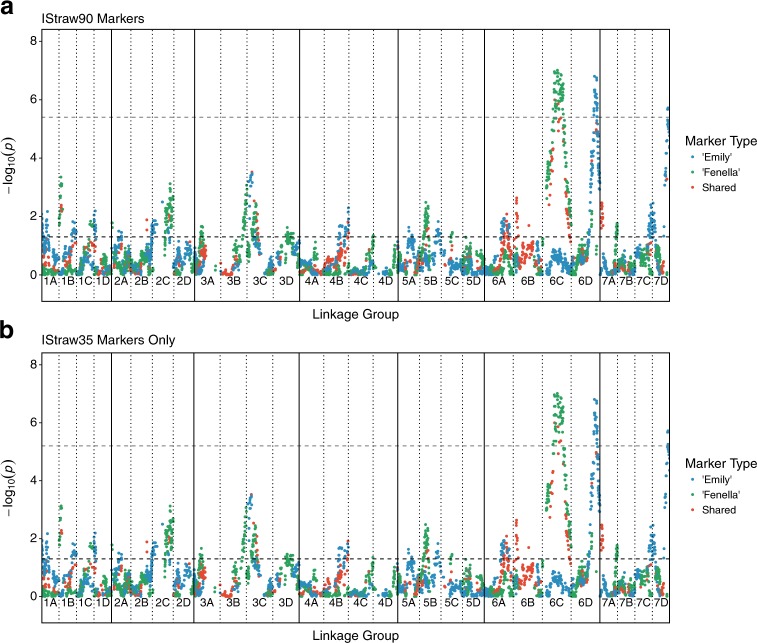
Kruskal–Wallis quantitative trait loci (QTL) analysis for ‘Emily’, ‘Fenella’ and shared markers across the 28 strawberry linkage groups (1A–7D). The coloured dots represent −log_10_(*p*) scores for the association of each single nucleotide polymorphism marker with resistance to *Phytophthora cactorum*, while the black dashed horizontal line represents the significance threshold 1.3 (*p* = 0.05) and the grey dashed horizontal line represents the Bonferroni correction significance threshold 5.4 and 5.2, for the IStraw90 markers (*p* = 4.31e−06) and IStraw35 markers (*p* = 5.99e−06), respectively. Regions on linkage groups 1A, 1B, 1C, 1D, 2A, 2B, 2C, 3A, 3B, 3C, 3D, 4B, 4C, 5A, 5B, 5C, 6A, 6B, 6C, 6D, 7A, 7C and 7D were significantly associated with resistance to *P. cactorum*, based on *p* < 0.05. **a** IStraw90 markers and **b** IStraw35 markers only

QTL mapping revealed 25 regions significantly associated with resistance to *P. cactorum* isolate P414, located on all seven chromosomes (*p* < 0.05; Fig. 1a and Table 1). The most significant marker associated with each region is shown in Table 1. Comparing the K–W analysis between the markers from the IStraw90 array and the subset on the IStraw35 array, the same 25 regions were still significant (*p* < 0.05; Fig. 1b and Table 1) before stepwise linear regression was performed. The identity of five focal points changed when using only the IStraw35 SNPs; LG1B, LG2B, LG3D, LG4B and LG5C (Table 1).

**Table 1:** *Phytophthora cactorum* resistance putative associated regions identified in the cultivated strawberry ‘Emily’ × ‘Fenella’ progeny by Kruskal–Wallis analysis using the Axiom^®^ IStraw90 and IStraw35 SNP arrays, *p* < 0.05

*Fragaria* × *ananassa* linkage group	Name of QTL	Position (bp)	Most significant SNP probe I90	Most significant SNP probe I35	Marker origin	*K* statistic	*p* Value	Significance^a^	Dominance	Retained after stepwise regression approach
LG1A		8,000,649	Affx-88889744	Affx-88889744	‘Emily’	7.3	0.0068	**	Dominant	No
LG1B		2,433,670/2,868,559	Affx-88809889	Affx-88869538	‘Fenella’	12.3/11.4	0.0004/0.0007	***	Dominant	Yes
LG1C		18,922,732	Affx-88814973	Affx-88814973	‘Fenella’	5.9	0.0145	*	Dominant	No
LG1D		1,698,614	Affx-88810385	Affx-88810385	‘Emily’	7.4	0.0065	**	Dominant	Yes
LG2A		617,651	Affx-88902526	Affx-88902526	‘Fenella’	5.7	0.0165	*	Dominant	No
LG2B		28,418,872/27,730,146	Affx-88830263	Affx-8883077	‘Emily’	5.5/4.5	0.0188/0.033	*	Dominant	Yes
LG2C		23,827,531	Affx-88827900	Affx-88827900	‘Fenella’	11.4	0.0008	***	Dominant	No
LG3A		9,963,752	Affx-88902698	Affx-88902698	‘Fenella’	5.3	0.0218	*	Dominant	Yes
LG3B		34,728,523	Affx-88844379	Affx-88844379	‘Fenella’	11.1	0.0009	***	Dominant	Yes
LG3C-A		11,900,442	Affx-88842594	Affx-88842594	‘Fenella’	8.5	0.0036	**	Dominant	Yes
LG3C-B		6,795,148	Affx-88836348	Affx-88836348	‘Emily’	12.8	0.0003	***	Dominant	Yes
LG3D		18,734,730/19,247,403	Affx-88831460	Affx-88831195	‘Fenella’	5.1/4.5	0.0238/0.0346	*	Dominant	No
LG4B		32,487,191/31,986,632	Affx-88851775	Affx-88858184	‘Emily’	7.8/5.9	0.0051/0.0152	*	Dominant	No
LG4C		32,798,311	Affx-88851534	Affx-88851534	‘Fenella’	4.0	0.0447	*	Dominant	No
LG5A		14,319,951	Affx-88867039	Affx-88867039	‘Emily’	5.1	0.0234	*	Dominant	No
LG5B		8,079,267	Affx-88862710	Affx-88862710	‘Fenella’	8.6	0.0033	**	Dominant	Yes
LG5C		14,681,019/14,693,803	Affx-88866823	Affx-88866815	‘Fenella’	4.4/4.4	0.0350/0.0350	*	Dominant	No
LG6A		29,917,230	Affx-88886294	Affx-88886294	Shared	11.8	0.0083	**	Dominant	Yes
LG6B		4,360,876	Affx-88816441	Affx-88816441	Shared	14.5	0.0023	**	Over-dominant (negative)	Yes
LG6C	*FaRPc6C*	20,125,920	Affx-88882258	Affx-88882258	‘Fenella’	28.4	9.77e−08	*******	Dominant	Yes
LG6D_A	*FaRPc6D*	31,199,915	Affx-88880166	Affx-88880166	‘Emily’	27.5	1.57e−07	******	Dominant	Yes
LG6D_B		27,650,927	Affx-88885149	Affx-88885149	‘Fenella’	7.4	0.0067	**	Dominant	No
LG7A		2,330,648	Affx-88894447	Affx-88894447	‘Fenella’	13.7	0.0034	**	Dominant	Yes
LG7C		21,270,542	Affx-88901970	Affx-88901970	‘Emily’	8.3	0.0040	**	Dominant	No
LG7D	*FaRPc7D*	20,941,169	Affx-88902178	Affx-88902178	‘Emily’	22.7	1.91e−06	*****	Dominant	Yes

^a^Significance value associated with the marker: ^*^0.05 > *p* > 0.01, ^**^0.01 > *p* > 0.001, ^***^0.001 > *p* > 0.0001, ^****^0.0001 > *p* > 0.00001, ^*****^0.00001 > *p* > 0.000001, ^******^0.00000.1 > *p* *>* 0.0000001 and ^*******^p < 0.0000001

### Fourteen putative associated regions remained after stepwise linear regression

Stepwise linear regression was performed and 11 of these regions were found to be non-significant, leaving 14 putatively associated regions with resistance to *P. cactorum* (Table S2). To increase the usefulness for downstream processes, the two adjacent SNPs in coupling phase either side of the focal resistance SNP for the 14 putative associated regions identified through the stepwise linear regression analysis are detailed in Table S3. Seven putatively associated regions were present in ‘Fenella’ only; located on LG1B, LG3A, LG3B, LG3C-A, LG5B, LG6C and LG7A (Table 1). Five putatively associated regions were present in ‘Emily’ only; located on LG1D, LG2B, LG3C-B, LG6D and LG7D (Table 1). Two putatively associated regions were present in both ‘Emily’ and ‘Fenella’, located LG6A and LG6B (Table 1). Of these significant putatively associated regions with resistance, three were identified on the A sub-genome (the most similar to *F. vesca*), five were identified on the B sub-genome (the most similar *F. iinumae*), three were identified on the C sub-genome and three were identified on the D sub-genome.

All but one of the putatively associated regions behaved in a dominant nature, LG6B was over-dominant for susceptibility (Table 1). If the individual was homozygous for either parent (AA/BB), it had a predicted crown rot disease score of 2.8/2.7. However, if the individual was heterozygous (AB/BA) at that locus then it had a predicted crown rot disease score of 3.4/3.2. The individuals that were heterozygous at this locus were more susceptible than either homozygous individuals.

### Detected putative QTL explain a large proportion of observed variation

**Fig. 2:**
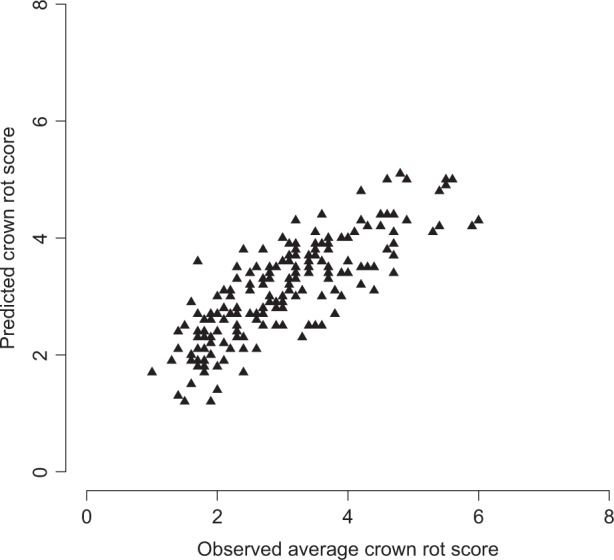
Correlation of predicted means and observed crown rot scores. Predicted means are highly positively correlated with observed average crown rot scores, *r*^2^ = 0.67

The linear regression also allowed the calculation of a contributing crown rot disease score to each of the 14 significant putatively associated regions with resistance (Table S2). The estimate of effect sizes were combined to produce each individual’s predicted score (Table S4). Individuals with no putatively associated regions with resistance were predicted to have a crown rot score of 5.5 (Table S2). The percentage effect of the 14 individual putatively associated regions ranged from 4.0 to 14.4% (Table S2). The three largest effect putatively associated regions, located on LG6C, LG6D_A and LG7D accounted for 10.3%, 14.4% and 11.8%, respectively and explained a total of 36.5% of the variation observed. The predicted means were plotted against observed average scores and were found to be positively correlated, *r*^2^ = 0.67 (Fig. 2).

The Bonferroni correction was calculated for both the IStraw90 map (0.05/11,598 = 4.31e−06) and the IStraw35 map (0.05/8,348 = 5.99e−06) and were plotted on the QTL analysis results as −log_10_(4.31e−06) and −log(5.99e−06), respectively (Figs. 1a, b, grey dashed lines). Looking at the analysis from both sets of markers, three putatively associated regions cross this threshold; LG6C, LG6D_A and LG7D (Fig. 1a, b). The three QTLs that passed the Bonferroni correction were named *FaRPc6C* (*Fragaria* × *ananassa* resistance to *P*. *cactorum* linkage group 6C), *FaRPc6D* and *FaRPc7D*, located on linkage groups 6C, 6D and 7D, respectively (Table 1).

rMQM analysis with markers Affx-88882258, Affx-88880166 and Affx-88902178 (*FaRPc6C*, *FaRPc6D* and *FaRPc7D*, respectively) selected as co-factors revealed no additional masked QTL.

### No epistatic interactions detected between three major QTL

Focusing on the three major effect QTL, ANOVA revealed there were no epistatic interactions between these QTL; *FaRPc6C*, *FaRPc6D* and *FaRPc7D*. Each QTL had an effect on its own and there were no pairwise interactions detected (Table S5). Levene’s test revealed that the requirement for homogeneity of variance was met for the three major QTL (*p* > 0.2761).

### Testing a subset of the mapping population progeny against a range of *P. cactorum* isolates revealed no significant interaction

A subset of 15 representative progeny from three sensitivities of the bi-parental cross; five lowly diseased individuals (EF011, EF021, EF101, EF147 and EF184) with average disease scores ranging from 1 to 2, five intermediate response individuals (EF041, EF060, EF141, EF164 and EF187), with average disease scores ranging from 2.7 to 4.3 and five highly diseased individuals (EF035, EF040, EF084, EF120 and EF166) with average disease scores ranging from 5.2 to 5.8, were tested for their response to three further isolates of *P. cactorum*, P404, P415 and P416 (Table 2). No major differences in host response were observed, individuals possessing few QTL were more susceptible than individuals possessing multiple QTL. However, some lines showed higher susceptibility than predicted (Table 2). Genotypes that possessed the three major effect QTL (*FaRPc6C*, *FaRPc6D* and *FaRPc7D*) or combinations of them were more resistant than those genotypes that did not possess any (Table 2).

**Table 2:** Evaluation of 15 representative individuals of the ‘Emily’ × ‘Fenella’ population and their resistance/susceptibility response to *Phytophthora cactorum* isolates, with presence/absence of resistance putatively associated regions detailed

Sensitivity	Individual	Predicted score	*Phytophthora cactorum* isolate				Presence/absence of resistance putative associated regions														Marker total	No. of major effect QTL
			P414	P404	P415	P416	*LG1B*	*LG1D*	*LG2B*	*LG3A*	*LG3B*	*LG3C-A*	*LG3C-B*	*LG5B*	*LG6A*	*LG6B*	*FaRPc6C*	*FaRPc6D*	*LG7A*	*FaRPc7D*		
Lowly diseased	EF147	1.2	1.9	2.6	2.7	1.4	0	1	0	1	1	1	0	1	1	1	**1**	**1**	1	**1**	11	3
	EF011	1.7	1.0	2.3	1.6	2.3	1	0	1	1	1	1	0	0	1	1	**1**	**1**	0	**1**	10	3
	EF101	1.9	1.6	2.6	2.8	2.0	0	1	0	0	0	1	0	1	1	1	**1**	**1**	1	**1**	9	3
	EF184	2.3	1.7	3.0	3.6	1.7	0	1	1	0	1	1	0	0	0	0	**1**	**1**	1	**1**	8	3
	EF021	2.6	1.9	2.9	2.3	4.0	1	1	0	1	1	0	1	1	0	0	**1**	**1**	0	0	8	2
Intermediate	EF164	2.5	3.0	3.8	2.7	4.0	1	1	1	1	0	0	0	1	0	0	0	**1**	1	**1**	8	2
	EF141	3.5	3.4	2.0	4.0	4.3	1	0	0	0	1	1	1	0	0	1	0	0	1	**1**	7	1
	EF041	3.6	4.0	3.6	3.4	3.7	0	1	0	0	1	0	1	1	0	0	0	0	1	**1**	6	1
	EF187	3.7	3.1	2.7	2.7	2.3	0	0	0	1	0	1	0	0	1	1	0	0	0	**1**	5	1
	EF060	4.3	3.2	3.2	2.0	2.7	1	0	0	0	0	0	0	1	0	0	**1**	0	1	0	4	1
Highly diseased	EF166	4.2	5.9	6.6	6.5	6.6	0	1	0	0	0	0	0	1	0	1	**1**	0	0	0	4	1
	EF120	4.3	6.0	6.4	6.4	6.6	0	1	1	0	0	1	1	0	0	1	0	0	0	0	5	0
	EF040	4.8	5.4	5.1	5.4	3.9	0	0	0	1	0	0	0	1	0	0	0	0	0	0	2	0
	EF035	5.0	5.5	5.8	5.3	4.0	0	0	0	0	1	0	0	1	0	0	0	0	0	0	2	0
	EF084	5.0	5.6	6.3	5.0	4.0	0	0	0	0	0	0	0	1	0	1	0	0	0	0	2	0

The presence of the major effect quantitative trait loci (QTL) *FaRPc6C*, *FaRPc6D* and *FaRPc7D* are highlighted in bold

ANOVA revealed there was no significant interaction (*p* = 0.0593) detected between the 15 representative progeny nested within the three sensitivities (lowly diseased, intermediate and highly diseased) and the four *P. cactorum* isolates tested (Table S6). There was a highly significant difference between the sensitivity groups (*p* < 2e−16) but not between *P. cactorum* isolates (*p* = 0.2046; Table S6).

### Preliminary GWAS revealed additional loci associated with resistance to *P. cactorum*

**Fig. 3:**
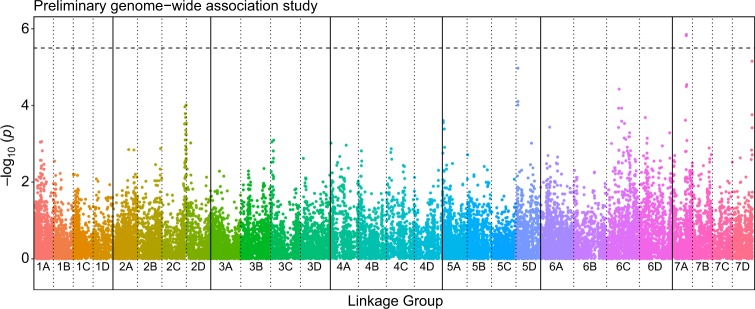
Manhattan plot of preliminary genome-wide association study on 114 strawberry accessions across the 28 strawberry linkage groups using PLINK^45^. The coloured dots represent −log_10_(*p*) scores for the analysis, while the black dashed horizontal line represents the significance threshold 5.5 (FDR BH *p* = 0.05). A region on linkage group 7A is significantly associated with resistance to *Phytophthora cactorum*

**Fig. 4:**
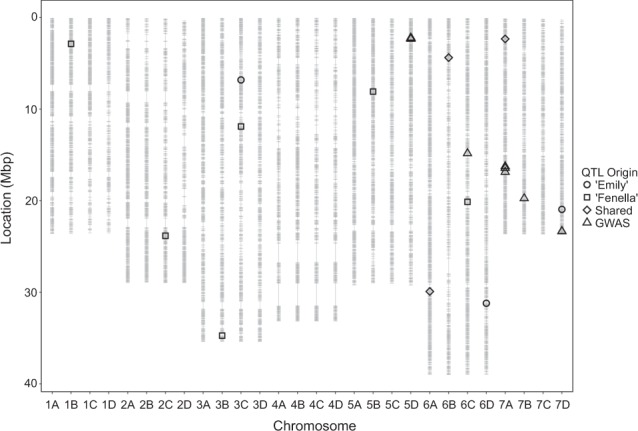
Hybrid consensus map depicting the position of *Phytophthora cactorum* putative resistance quantitative trait loci (QTL) identified from the ‘Emily’ × ‘Fenella’ progeny and the preliminary genome-wide association study (GWAS). The 35,154 markers in Mbp (grey lines) from the combined map of the five bi-parental crosses, for the 28 linkage groups of octoploid strawberry (1A-7D) were scaled to the *Fragaria vesca* genome v2.0^22^. Locations of putative QTL originating from ‘Emily’ (circles), locations of putative QTL originating from ‘Fenella’ (squares), locations of putative QTL shared in both cultivars (diamonds) and locations of putative QTL identified from the preliminary GWAS (triangles) are shown

A preliminary GWAS identified two SNPs using PLINK, significantly associated with resistance to *P. cactorum* on linkage group 7A (FDR BH *p* < 0.05; Table 3 and Fig. 3). A further two markers were identified that are of potential interest, located on linkage groups 5D and 7D (Table 3 and Fig. 3). None of these markers overlapped with the QTL discovered in the bi-parental cross (Fig. 4), although all linkage groups except 5D had putatively associated regions identified on them. The SNP identified on LG7D (Affx-88900641) was located within the QTL region of *FaRPc2* identified by Mangandi et al.^19^, based on its position on the *F. vesca* Hawaii 4 v1.1 genome^47^.

**Table 3:** Details of the most significant single nucleotide polymorphism (SNP) markers associated with resistance to *Phytophthora cactorum* identified in the preliminary genome-wide association study of 114 individuals, identified by both PLINK^45^ and TASSEL^46^ analyses

*Fragaria* *×* *ananassa* linkage group	Position on *Fragaria vesca* v1.1 (bp)	Most significant SNP probe	PLINK^45^		TASSEL^46^		IStraw35 marker?
			Raw *p* value	FDR BH *p* value	Raw *p* value	FDR BH *p* value	
LG5D	2 411 572	Affx-88859864	1.06e−05	0.0956	3.24e−06	0.0673	No
LG7A	14 759 809	Affx-88897773	1.51e−06	0.0271	2.89e−05	0.1708	Yes
	14 946,159	Affx-88897860	1.41e−06	0.0271	2.97e−06	0.0673	Yes
LG7D	19,557,035	Affx-88900641	6.98e−06	0.0839	9.72e−05	0.2734	Yes

Raw *p* values and false-discovery rate Benjamini–Hochberg (FDR BH) *p* values are shown

### Presence of known classes of resistance genes in and around QTL regions

Focusing on the three major effect QTL (*FaRPc6C*, *FaRPc6D* and *FaRPc7D*) identified from the bi-parental cross, at least one NLR gene was identified within 1 Mbp either side of the most significant SNP, located on the *F. vesca* Hawaii 4 v1.1 genome^47^ (Table 4). In addition at least one RLK was also identified around each focal SNP, as well as six RLP genes around *FaRPc6D* (Table 4).

**Table 4:** Details of the number of nucleotide-leucine rich repeat (NLR) receptors, receptor-like kinases (RLK) and receptor-like proteins (RLP) within 1 Mbp either side of the most significant marker on *Fragaria vesca* Hawaii 4 v1.1 for the quantitative trait loci (QTL) identified in the bi-parental cross and preliminary genome-wide association study (GWAS)

	Name/linkage group	Most significant SNP marker	Location on *Fragaria vesca* v1.1 (bp)	Number of genes within 1 Mbp either side of most significant SNP in *Fragaria vesca* v1.1		
				NLR	RLK	RLP
Major effect QTL from bi-parental cross	*FaRPc6C*	Affx-88882258	21,039,403	1	5	0
	*FaRPc6D*	Affx-88880166	15,257,362	1	1	6
	*FaRPc7D*	Affx-88902178	21,972,447	6	9	0
Putative QTL from preliminary GWAS	LG5D	Affx-88859864	2,411,572	4	10	1
	LG7A	Affx-88897773	14,759,809	7	8	2
		Affx-88897860	14,946,159	10	8	2
	LG7D	Affx-88900641	19,557,035	15	12	1

In the preliminary GWAS, the SNP of potential interest on LG5D is located within an RLK on the *F. vesca* Hawaii 4 v1.1 genome^47^ (Table 4). The two significant SNPs identified on LG7A were close together and there was an RLK gene between them (Affx-88897773 and Affx-88897860). The SNP of potential interest on LG7D was also located within an RLK. Around all of these SNPs there were multiple NLR genes and several RLP genes located on the *F. vesca* Hawaii 4 v1.1 genome^47^ (Table 4).

## Discussion

Resistance to *P. cactorum* in octoploid strawberry is known to be under complex genetic control, with multiple QTL involved in resistance^16,18^. In this study, a total of 14 putative associated regions with resistance to crown rot were identified using stepwise linear regression, of which three were found to still be significant after Bonferroni correction (*FaRPc6C*, *FaRPc6D* and *FaRPc7D*). The two approaches resulted in a different number of final putative associated regions, this was due to the Bonferroni correction using a genome-wide threshold for significant markers compared to the stepwise linear regression method which allowed the highest associations that passed single marker-based thresholds.

In a previous study, five putative QTLs were identified in the *F.* *×* *ananassa* ‘Capitola’ *×* ‘CF1116’ progeny, with the QTL effects ranging from 6.5 to 10.2% and coming from both parents^16^. Our results are comparable, with the QTL identified from both parents and with effect sizes ranging from 4.0 to 14.4%. The linkage maps produced by AFLP, SSR and SCAR (sequence characterised amplified region) markers of ‘Capitola’ and ‘CF1116’ were resolved into 47 and 45 linkage groups for the female and male maps, respectively^16^. Due to the unavailability of published markers we were unable to verify if any of the identified QTL were the same as the QTL identified in this study.

Comparing the QTL analysis of the bi-parental cross using markers from the IStraw90 and IStraw35 arrays, there were no differences between the putative associated regions identified, just a difference between the identity of the focal SNP for five linkage groups (LG1B, LG2B, LG3D, LG4B and LG5C). The informative markers from this bi-parental cross were used in the development of the IStraw35 array, along with informative markers from many other bi-parental crosses^30^. The cheaper IStraw35 array provides sufficient markers to identify *P. cactorum* resistance QTL within the bi-parental cross as well as in the preliminary GWAS, as most of the IStraw90 markers were present on the IStraw35 array.

*R* genes effective against *Phytophthora* spp. typically contain NLR domains, which can directly or indirectly perceive pathogen effectors^48^. NLR genes are abundant in most plant genomes and are involved in the detection of a wide range of pathogens; oomycetes, fungi, viruses, bacteria, nematodes and insects. The diploid *F. vesca* has been well studied and at least 144 NLR genes have been identified^49^. Along with this class of resistance gene, RLK and RLP genes have been associated with resistance to *Phytophthora* pathogens^29–33^. Disease resistance genes are often found in clusters in the genome^50^ and we observed multiple genes of each resistance class around our QTL regions in *F. vesca*. Further work is required to determine which of the genes are responsible for the resistance. The release of a more comprehensive *F.* *×* *ananassa* genome will aid this.

No race structure has been reported for *P. cactorum* and our results investigating progeny–isolate interactions revealed no significant interaction. However, further investigation into the strawberry: *P. cactorum* pathosystem is required to explore thoroughly whether there is a race structure present that corresponds to the quantitative, putatively, *R* gene resistance that we have described. Individuals that possessed the three major QTL were more resistant to all four isolates of *P. cactorum* than individuals that possessed fewer or no QTL, indicating that the resistance identified in the ‘Emily’ *×* ‘Fenella’ population would be useful against different isolates of *P. cactorum*. Several of the 11 other putative QTL, are of too small effect size for breeders to utilise in a marker-assisted breeding approach. However, if the three major effect QTL (combined score reduction of 2) could be introgressed into elite strawberry germplasm, then a good base level of resistance to *P. cactorum* could be provided, with other putative QTL, such as LG3A (score reduction of 0.4 and effect size of 7.7%) supplementing them. Interestingly ‘Emily’ possesses two of the major effect QTL (*FaRPc6D* and *FaRPc7D*), but is more susceptible than ‘Fenella’. Possibly there are additional undetected susceptibility factors affecting the overall resistance/susceptibility status of the plant that are homozygous and therefore contain unmappable loci. Similarly, some genotypes showed higher susceptibility than predicted (EF120 and EF167) indicating that additional undetected factors may affect the expression of some QTL. There may also be undiscovered recessively inherited resistance loci that are undetectable as they only segregate in one parent.

In *F. vesca* a single major gene locus was identified on the proximal end of LG6, named *RPc-1* (Resistance *to Phytophthora cactorum 1*). *RPc-1* explained 74.4% of the variation observed and was identified as spanning a region of 801 genes and 69 potential plant disease resistance genes, including multiple different classes of resistance gene, in a 3.3 Mb region from position 5,151,532 to 9,201,791 bp^51^. *RPc-1* would be on *F.* *×* *ananassa* LG6A, based on the convention of van Dijk et al.^24^. In the bi-parental cross a minor putatively associated region was identified on LG6A; the corresponding genomic location in *F. vesca* is distant, ~29 Mbp upstream of *RPc-1*. Toljamo et al. observed the expression of potential resistance genes in the *RPc-1* region, in *P. cactorum* inoculated *F. vesca* Hawaii 4 roots^52^. Within the *RPc-1* locus, four NLR genes (101,306,457; 101,297,569; 101,300,750 and 101,304,699) were identified as being expressed, two of which were significantly down-regulated within the inoculated plants (101,300,750 and 101,304,699)^52^. The authors propose 101,297,569 as a strong candidate as it had the highest expression (mean expression level, 32.41) of the NLR genes in that region. Other types of resistance genes were identified within the *RPc-1* locus, two l-type-lectin-RLKs were significantly upregulated (101,310,048 and 101,309,756; log_2_ fold change 3.50 and 2.25, respectively), these were both considered strong candidates. Two G-type-lectin-RLKs (101,305,393 and 101,305,094) and one RLP (101,290,881) were also upregulated in the inoculated roots^52^. Our results are comparable to the study in *F. vesca*, as we identified multiple NLR and RLK candidates nearby focal SNPs of each QTL. Further characterisation is required to fully understand the resistance mechanisms against *P. cactorum* in *F. vesca*, as well as *F*. *×* *ananassa*.

Two of our major effect QTL were identified on the D sub-genome (*FaRPc6D* and *FaRPc7D*), which is the least similar to *F. vesca*^24^. Recently, a major locus associated with resistance to *P. cactorum* in *F*. *×* *ananassa* was identified on LG7D, named *FaRPc2*^19^. The small GWAS of the wider germplasm highlighted different potential areas of the genome associated with resistance to *P. cactorum*, compared to the putative QTL identified from the bi-parental cross, indicating further loci, not captured in our study of ‘Emily’ and ‘Fenella’ to be exploited for resistance. A possible reason for none of the QTL overlapping between the bi-parental cross and the preliminary GWAS is that it included a much more diverse range of material not highly related to either parent and therefore the resistance present in ‘Emily’ and ‘Fenella’ may not be present at a high enough frequency to be detected. Alternatively, the markers present on the IStraw35 array may not be in linkage disequilibrium with the QTL and therefore have little power to detect QTL in an association test. The SNP identified on LG7D in the preliminary GWAS was in the same region as the major locus *FaRPc2*, identified by Mangandi et al.^19^, when mapped to the *F. vesca* Hawaii 4 v1.1 genome^47^, indicating that the *FaRPc2* resistance loci may be present in our germplasm. In the same study, further QTL on LGs 1D, 3B, 5B, 6A and 6B were also identified, however, they were not consistent across the replicates or significant enough to be confirmed^19^.

Two approaches (PLINK and TASSEL) were used to analyse the preliminary GWAS data, since it was underpowered due to the small number of individuals investigated. However, despite it being underpowered, several significant SNPs were identified using PLINK and further potential SNPs were highlighted using both PLINK and TASSEL. Overall, there was good concordance between the two approaches, but the SNPs were more significant using PLINK, compared to TASSEL. Further individuals are required to be tested to increase the power of the analysis.

Quantitative resistance provides many challenges to breeders due to the complex nature of inheritance. However, this complexity can increase the durability of resistance as it might be harder for the pathogen to overcome^53^. To a large extent, the ‘Emily’ *×* ‘Fenella’ progeny responded similarly to each of the isolates tested (though there is some slight indication of variation in some *P. cactorum* isolates to the same plant genotypes), signifying that the three major effect QTL would be useful in commercial cultivars to provide broad-spectrum partial resistance using marker-assisted breeding. In order to utilise the full spectrum of the identified resistance, genomic selection approaches may prove useful, though the material in this study is not of sufficient commercial quality to warrant this approach for the bi-parental population. In future studies, it would be useful to explore the resistance in the wider germplasm, using a larger GWAS approach on many hundreds of accessions, using the same isolate, though it is possible, given the poor transferability of markers, that alternative SNP arrays or genotyping approaches may be needed to realise the full power of this approach. This would provide more information about the status of resistance within the population and identify parents with other desirable traits. Subsequent work is also required to identify the gene(s) within the QTL regions and elucidate the mechanism of resistance. Future work will address whether the quantitative resistance to *P*. *cactorum* is a combination of multiple quantitative gene-for-gene interactions between the host and the pathogen by studying both the host and the pathogen in greater detail.

## Supplementary information

Figure S1

Figure S2

Table S1

Table S2

Table S3

Table S4

Table S5

Table S6

Dataset 1

Dataset 2

## References

[1] Hummer Kim E., Hancock James (2009). Strawberry Genomics: Botanical History, Cultivation, Traditional Breeding, and New Technologies. Genetics and Genomics of Rosaceae.

[2] Maas JL (2014). Strawberry diseases and pests—progress and problems. Acta Hortic..

[3] Martínez F, Castillo S, Carmona E, Avilés M (2010). DIssemination of *Phytophthora cactorum*, cause of crown rot in strawberry, in open and closed soilless growing systems and the potential for control using slow sand filtration. Sci. Hortic..

[4] Erwin, D. C., Ribeiro, O. K. *Phytophthora Diseases Worldwide*. (American Phytopathological Society (APS Press), St. Paul, Minnesota, USA, 1996).

[5] Deutschmann VF (1954). Eine Wurzelfäule an Erdbeeren, hervorgerufen durch *Phytophthora cactorum* (Leb. et Cohn) Schröt. Nachr. Des. Dtsch. Pflanzenschutzd..

[6] Rose DH (1924). Leather rot of strawberries. J. Agric. Res..

[7] Stensvand A, Herrero ML, Talgø V (1999). Crown rot caused by *Phytophthora cactorum* in Norwegian strawberry production. EPPO Bull..

[8] Ellis MA, Grove GG (1983). Leather rot in Ohio strawberries. Plant Dis..

[9] Eikemo H (2004). Genetic variation between *Phytophthora cactorum* isolates differing in their ability to cause crown rot in strawberry. Mycol. Res..

[10] Eikemo H, Stensvand A (2015). Resistance of strawberry genotypes to leather rot and crown rot caused by *Phytophthora cactorum*. Eur. J. Plant Pathol..

[11] Maas, J. L. *Compendium of Strawberry Diseases*. 2nd ed. (The American Phytopathological Society, Minnesota, USA, 1998).

[12] Harris DC, Stickels JE (1981). Crown rot (*Phytophthora cactorum*) in glasshouse-grown strawberries at East Malling Research Station. Plant Pathol..

[13] Khew KL, Zentmyer GA (1973). Chemotactic response of zoospores of five species of Phytophthora. Phytopathology.

[14] Hardham AR (2001). The cell biology behind *Phytophthora* pathogenicity. Austral Plant Pathol..

[15] Vleeshouwers VGAA OliverRP (2014). Effectors as tools in disease resistance breeding against biotrophic, hemibiotrophic, and necrotrophic plant pathogens. Mol. Plant Microbe Interact..

[16] Denoyes-Rothan B (2004). QTL analysis for resistances to *Colletotrichum acutatum* and *Phytophthora cactorum* in octoploid strawberry (*Fragaria* x *ananassa*). Acta Hortic..

[17] Shaw DV, Hansen J, Browne GT (2006). Genotypic variation for resistance to *Phytophthora cactorum* in a California strawberry breeding population. J. Am. Soc. Hortic. Sci..

[18] Shaw DV, Hansen J, Browne GT, Shaw SM (2008). Components of genetic variation for resistance of strawberry to *Phytophthora cactorum* estimated using segregating seedling populations and their parent genotypes. Plant Pathol..

[19] Mangandi J (2017). Pedigree-based analysis in a multiparental population of octoploid strawberry reveals QTL alleles conferring resistance to *Phytophthora cactorum*. Genes Genomes Genet.

[20] Harrison RE, Luby JJ, Furnier GR, Hancock JF, Cooley D (1998). Variation for susceptibility to crown rot and powdery mildew in wild strawberry from North America. Acta Hortic..

[21] Hummer, K. E., Bassil, N., Njuguna, W. *Fragaria*. (Springer, Berlin, Heidelberg, 2011), pp 17–44.

[22] Tennessen JA, Govindarajulu R, Ashman TL, Liston A (2014). Evolutionary origins and dynamics of octoploid strawberry subgenomes revealed by dense targeted capture linkage maps. Genome Biol. Evol..

[23] Hirakawa H (2014). Dissection of the octoploid strawberry genome by deep sequencing of the genomes of *Fragaria* species. DNA Res..

[24] van Dijk T (2014). Genomic rearrangements and signatures of breeding in the allo-octoploid strawberry as revealed through an allele dose based SSR linkage map. BMC Plant Biol..

[25] Roach JA, Verma S, Peres NA, Jamieson AR, Weg WE (2016). Bink MCAM et al. *FaRXf1*: a locus conferring resistance to angular leaf spot caused by *Xanthomonas fragariae* in octoploid strawberry. Theor. Appl. Genet..

[26] Gezan SA, Osorio LF, Verma S, Whitaker VM (2017). An experimental validation of genomic selection in octoploid strawberry. Hortic. Res..

[27] Verma S (2017). Clarifying sub-genomic positions of QTLs for flowering habit and fruit quality in U.S. strawberry (*Fragaria* × *ananassa*) breeding populations using pedigree-based QTL analysis. Hortic. Res..

[28] Bassil, N. V. et al. Development and preliminary evaluation of a 90 K Axiom® SNP array for the allo-octoploid cultivated strawberry *Fragaria* × *ananassa*. *BMC Genom.* **16**, 155 (2015). 10.1186/s12864-015-1310-1 PMC4374422 25886969

[29] Verma S, Whitaker V (2016). A new technology enabling new advances in strawberry genetics. J. Hortic..

[30] Verma, S. et al. Development and evaluation of the Axiom^®^ IStraw35 384HT array for the allo-octoploid cultivated strawberry *Fragaria* × *ananassa*. *Acta Hortic*. **1156**, 75–82 (2017).

[31] Kamoun S (2014). The top 10 oomycete pathogens in molecular plant pathology. Mol. Plant Pathol..

[32] Vleeshouwers (2011). Understanding and exploiting late blight resistance in the age of effectors. Ann. Rev. Phytopathol..

[33] Armitage AD (2018). Bioinformatic characterisation of the effector repertoire of the strawberry pathogen *Phytophthora cactorum*. PLoS One.

[34] Wang Y, Bouwmeester K, Beseh P, Shan W, Govers F (2014). Phenotypic analyses of *Arabidopsis* T-DNA insertion lines and expression profiling reveal that multiple L-type lectin receptor kinases are involved in plant immunity. Mol. Plant Microbe Interact..

[35] Bouwmeester K (2011). The lectin receptor kinase LecRK-I.9 is a novel phytophthora resistance component and a potential host target for a RXLR effector. PLoS Pathog.

[36] Bouwmeester K (2014). The Arabidopsis lectin receptor kinase LecRK-I.9 enhances resistance to *Phytophthora infestans* in Solanaceous plants. Plant Biotechnol. J..

[37] Du Y, Berg J, Govers F, Bouwmeester K (2015). Immune activation mediated by the late blight resistance protein R1 requires nuclear localization of R1 and the effector AVR1. New Phytol..

[38] Blair JE, Coffey MD, Park SY, Geiser DM, Kang S (2008). A multi-locus phylogeny for *Phytophthora* utilizing markers derived from complete genome sequences. Fungal Genet. Biol..

[39] Whitehouse AB, Govan CL, Hammond KJ, Sargent DJ, Simpson DW (2011). Meristem culture for the elimination of the strawberry crown rot pathogen *Phytophthora cactorum*. J. Berry Res.

[40] Bell JA, Simpson DW, Harris DC (1997). Development of a method for screening Strawberry germplasm for resistance to *Phytophthora cactorum*. Acta Hortic..

[41] Team RC. *R: A Language and Environment for Statistical Computing*. (R Foundation for Statistical Computing, Vienna, Austria, 2015) https://www.R-project.org/.

[42] Vickerstaff, R. J., Harrison, R. J. *Crosslink: A Fast, Scriptable Genetic Mapper for Outcrossing Species*. 10.1101/135277.

[43] Cockerton HM (2018). Identification of powdery mildew resistance QTL in strawberry (*Fragaria* × *ananassa*). Theor. Appl. Genet.

[44] van Ooijen, J. W. *MapQTL*^*®*^*5*. (Kyazma B. V., Wageningen, Netherlands, 2004).

[45] Purcell S (2007). PLINK: a tool set for whole-genome association and population-based linkage analyses. Am. J. Hum. Genet..

[46] Bradbury PJ (2007). TASSEL: software for association mapping of complex traits in diverse samples. Bioinformatics.

[47] Shulaev V (2011). The genome of woodland strawberry (*Fragaria vesca*). Nat. Genet..

[48] Dangl JL, Jones JDG (2001). Plant pathogens and integrated defence responses to infection. Nature.

[49] Zhong Y, Yin H, Sargent DJ, Malnoy M, Cheng ZMM (2015). Species-specific duplications driving the recent expansion of NBS-LRR genes in five Rosaceae species. BMC Genom..

[50] Michelmore RW, Meyers BC (1998). Clusters of resistance genes in plants evolve by divergent selection and a birth-and-death process. Genome Res..

[51] Davik J (2015). A ddRAD based linkage map of the cultivated strawberry, *Fragaria xananassa*. PLoS One.

[52] Toljamo Anna, Blande Daniel, Kärenlampi Sirpa, Kokko Harri (2016). Reprogramming of Strawberry (Fragaria vesca) Root Transcriptome in Response to Phytophthora cactorum. PLOS ONE.

[53] Brun H (2010). Quantitative resistance increases the durability of qualitative resistance to *Leptosphaeria maculans* in *Brassica napus*. New Phytol..

