# Supplementary figures and images for: Quantitative trait loci controlling Phytophthora cactorum resistance in the cultivated octoploid strawberry (Fragaria × ananassa)
Source: Hortic Res. 2019 May 1;6:60. doi: 10.1038/s41438-019-0136-4 (PMC6491645; doi:10.1038/s41438-019-0136-4)

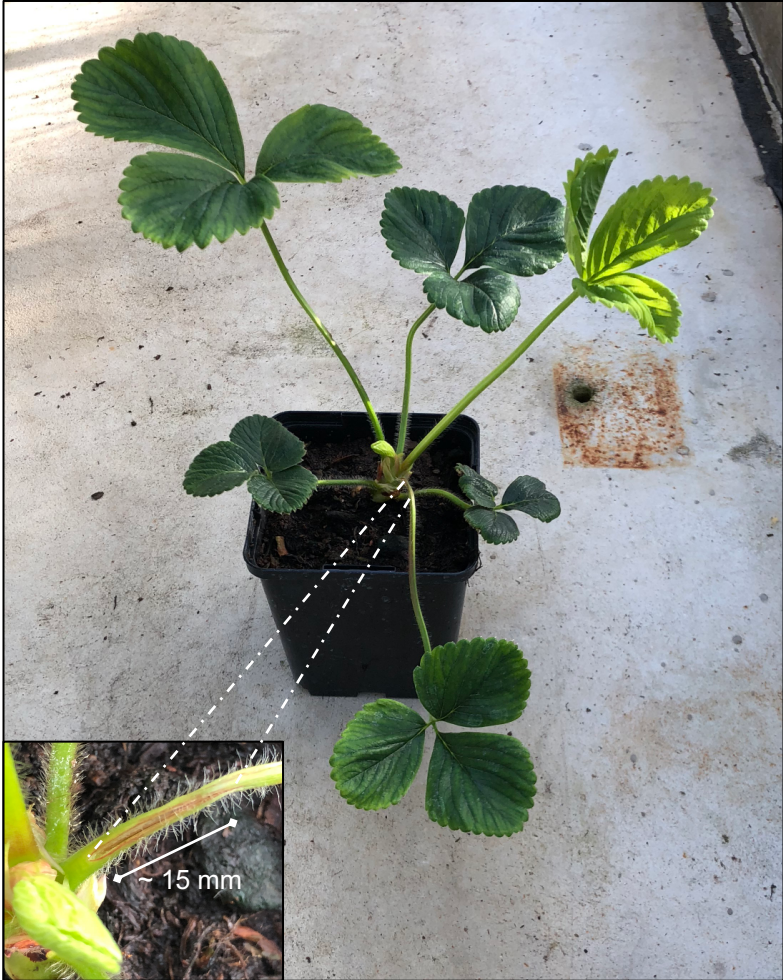

Supplement: Supplementary file 1 — Figure S1 [file 41438_2019_136_MOESM1_ESM.pdf]

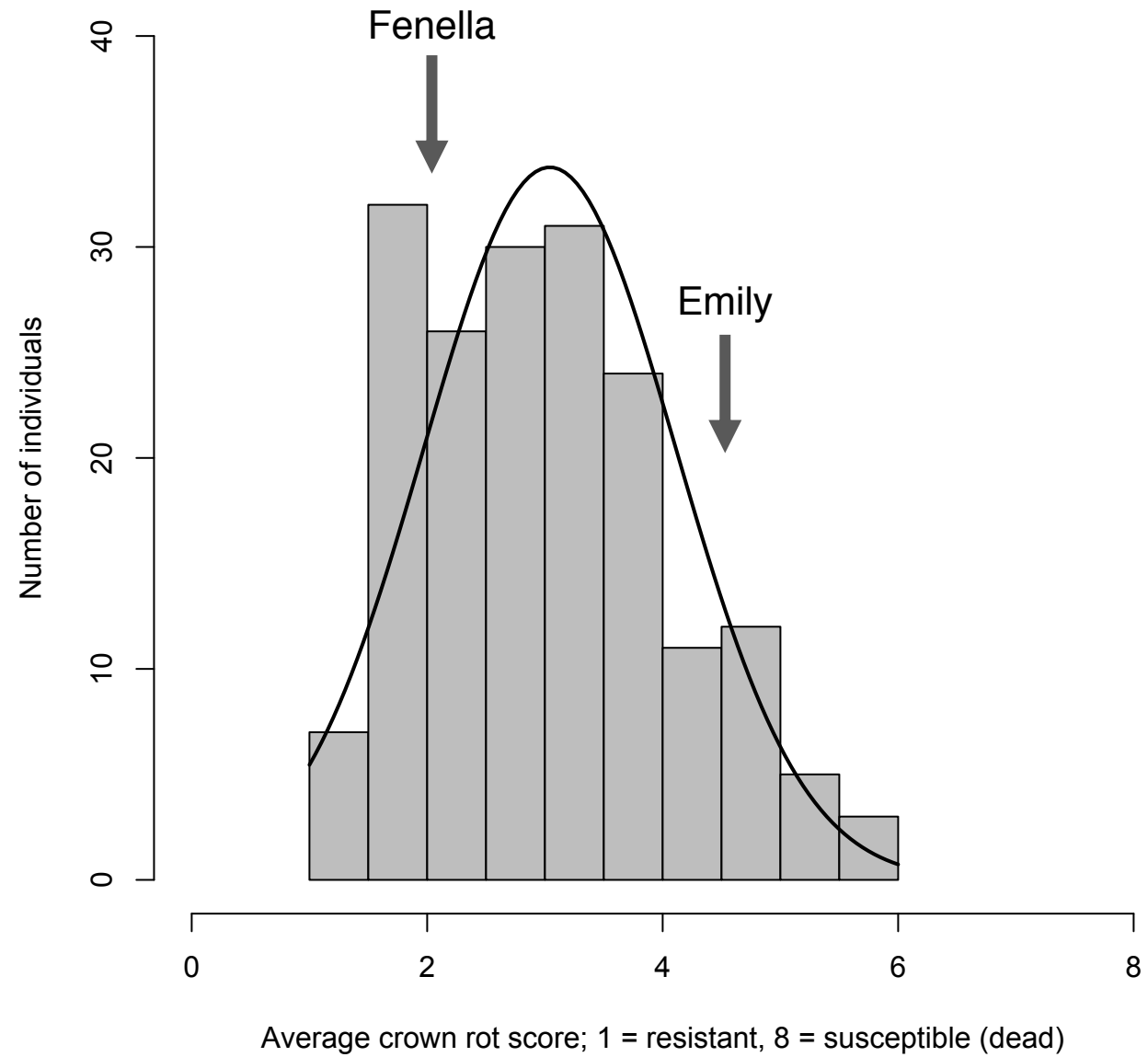

Supplement: Supplementary file 2 — Figure S2 [file 41438_2019_136_MOESM2_ESM.pdf]
