# Supplementary material for: Quantitative trait loci controlling Phytophthora cactorum resistance in the cultivated octoploid strawberry (Fragaria × ananassa)
Source: Hortic Res. 2019 May 1;6:60. doi: 10.1038/s41438-019-0136-4 (PMC6491645; doi:10.1038/s41438-019-0136-4)
Supplement: Supplementary file 4 — Table S2 [file 41438_2019_136_MOESM4_ESM.docx]

**Table S2.** Stepwise linear regression analysis estimated the effect of each *Phytophthora cactorum* resistance putative associated region from the cultivated strawberry ‘Emily’ x ‘Fenella’ progeny.

| **Parameter** | **Name of**  **QTL** | **Estimate of effect size** | **s.e.** | ***t* pr.** | **Significance^a^** | **Percentage**  **effect** |
| --- | --- | --- | --- | --- | --- | --- |
| **Constant** |  | 5.524 | 0.150 | <2e-16 | *** |  |
| ***LG1B*** |  | -0.317 | 0.100 | 0.002 | ** | -5.7% |
| ***LG1D*** |  | -0.278 | 0.010 | 0.006 | ** | -5.0% |
| ***LG2B*** |  | -0.221 | 0.096 | 0.022 | * | -4.0% |
| ***LG3A*** |  | -0.424 | 0.097 | 2.11e-05 | *** | -7.7% |
| ***LG3B*** |  | -0.242 | 0.099 | 0.015 | * | -4.4% |
| ***LG3C-A*** |  | -0.241 | 0.097 | 0.014 | * | -4.4% |
| ***LG3C-B*** |  | -0.239 | 0.099 | 0.017 | * | -4.3% |
| ***LG5B*** |  | -0.289 | 0.096 | 0.003 | ** | -5.4% |
| ***LG6A*** |  | -0.298 | 0.097 | 0.002 | ** | -5.4% |
| ***LG6B*** |  | -0.235 | 0.099 | 0.019 | * | -4.3% |
| ***LG6C*** | ***FaRPc6C*** | -0.571 | 0.098 | 3.11e-08 | *** | -10.3% |
| ***LG6D-A*** | ***FaRPc6D*** | -0.794 | 0.097 | 8.40e-14 | *** | -14.4% |
| ***LG7A*** |  | -0.251 | 0.099 | 0.013 | * | -4.5% |
| ***LG7D*** | ***FaRPc7D*** | -0.654 | 0.098 | 3.66e-10 | *** | -11.8% |

^a^ Significance value associated with the marker: *0.05>*p*>0.01 **0.01>*p*>0.001 ****p*<0.0001
