# Supplementary material for: Quantitative trait loci controlling Phytophthora cactorum resistance in the cultivated octoploid strawberry (Fragaria × ananassa)
Source: Hortic Res. 2019 May 1;6:60. doi: 10.1038/s41438-019-0136-4 (PMC6491645; doi:10.1038/s41438-019-0136-4)
Supplement: Supplementary file 5 — Table S3 [file 41438_2019_136_MOESM5_ESM.docx]

**Table S3**. Details of the two adjacent markers in coupling phase, either side of the most significant single nucleotide polymorphism (SNP) marker (focal SNP shown in bold) for the 14 putative associated regions.

| **Putative QTL** | **SNP marker** | **Position  (bp)** | **Marker  type** | **Phase** | **Maternal** | **Paternal** |
| --- | --- | --- | --- | --- | --- | --- |
| ***FaRPc6C*** | Affx-88882160 | 19 964 587 | nnxnp | {-1} | nn | np |
|  | Affx-88882239 | 20 103 160 | nnxnp | {-1} | nn | np |
|  | **Affx-88882258** | **20 125 921** | **nnxnp** | **{-1}** | **nn** | **np** |
|  | Affx-88882267 | 20 164 065 | nnxnp | {-1} | nn | np |
|  | Affx-88882328 | 20 334 955 | nnxnp | {-1} | nn | np |
| ***FaRPc6D*** | Affx-88880086 | 31 053 052 | lmxll | {1-} | lm | ll |
|  | Affx-88880150 | 31 179 546 | lmxll | {1-} | lm | ll |
|  | **Affx-88880166** | **31 199 915** | **lmxll** | **{1-}** | **lm** | **ll** |
|  | Affx-88880191 | 31 251 305 | lmxll | {1-} | lm | ll |
|  | Affx-88880248 | 31 364 105 | lmxll | {1-} | lm | ll |
| ***FaRPc7D*** | Affx-88902364 | 20 616 478 | lmxll | {0-} | lm | ll |
|  | Affx-88902199 | 20 882 292 | lmxll | {0-} | lm | ll |
|  | **Affx-88902178** | **20 941 169** | **lmxll** | **{0-}** | **lm** | **ll** |
|  | Affx-88902169 | 20 961 449 | lmxll | {0-} | lm | ll |
|  | Affx-88902134 | 21 040 377 | lmxll | {0-} | lm | ll |
| ***LG1B*** | Affx-88809929 | 2 392 900 | nnxnp | {-0} | nn | np |
|  | Affx-88809928 | 2 393 174 | nnxnp | {-0} | nn | np |
|  | **Affx-88809889** | **2 433 670** | **nnxnp** | **{-0}** | **nn** | **np** |
|  | Affx-88809884 | 2 438 204 | nnxnp | {-0} | nn | np |
|  | Affx-88809880 | 2 445 735 | nnxnp | {-0} | nn | np |
| ***LG1D*** | Affx-88810467 | 1 587 962 | lmxll | {0-} | lm | ll |
|  | Affx-88810429 | 1 629 606 | lmxll | {0-} | lm | ll |
|  | **Affx-88810385** | **1 698 614** | **lmxll** | **{0-}** | **lm** | **ll** |
|  | Affx-88810316 | 1 798 708 | lmxll | {0-} | lm | ll |
|  | Affx-88810101 | 2 188 276 | lmxll | {0-} | lm | ll |
| ***LG2B*** | Affx-88830297 | 28 383 500 | lmxll | {0-} | lm | ll |
|  | Affx-88830287 | 28 391 110 | lmxll | {0-} | lm | ll |
|  | **Affx-88830263** | **28 418 872** | **lmxll** | **{0-}** | **lm** | **ll** |
|  | Affx-88830181 | 28 577 116 | lmxll | {0-} | lm | ll |
|  | Affx-88830139 | 28 636 758 | lmxll | {0-} | lm | ll |
| ***LG3A*** | Affx-88843279 | 9 662 171 | nnxnp | {0-} | nn | np |
|  | Affx-88843282 | 9 662 742 | nnxnp | {0-} | nn | np |
|  | **Affx-88902698** | **9 963 752** | **nnxnp** | **{0-}** | **nn** | **np** |
|  | Affx-88836733 | 10 238 807 | nnxnp | {0-} | nn | np |
|  | Affx-88866192 | 10 828 667 | nnxnp | {0-} | nn | np |
| ***LG3B*** | Affx-88844243 | 34 310 744 | nnxnp | {0-} | nn | np |
|  | Affx-88844250 | 34 321 101 | nnxnp | {0-} | nn | np |
|  | **Affx-88844379** | **34 728 523** | **nnxnp** | **{0-}** | **nn** | **np** |
|  | Affx-88844401 | 34 755 453 | nnxnp | {0-} | nn | np |
|  | Affx-88844408 | 34 762 966 | nnxnp | {0-} | nn | np |
| ***LG3C-A*** | Affx-88842759 | 11 620 632 | nnxnp | {0-} | nn | np |
|  | Affx-88842756 | 11 621 946 | nnxnp | {0-} | nn | np |
|  | **Affx-88842594** | **11 900 442** | **nnxnp** | **{0-}** | **nn** | **np** |
|  | Affx-88842586 | 11 905 296 | nnxnp | {0-} | nn | np |
|  | Affx-88842561 | 11 928 771 | nnxnp | {0-} | nn | np |
| ***LG3C-B*** | Affx-88836462 | 6 572 905 | lmxll | {0-} | lm | ll |
|  | Affx-88903415 | 6 788 989 | lmxll | {0-} | lm | ll |
|  | **Affx-88836348** | **6 795 148** | **lmxll** | **{0-}** | **lm** | **ll** |
|  | Affx-88836176 | 7 064 274 | lmxll | {0-} | lm | ll |
|  | Affx-88842521 | 11 975 954 | lmxll | {0-} | lm | ll |
| ***LG5B*** | Affx-88862791 | 7 992 628 | nnxnp | {0-} | nn | np |
|  | Affx-88862755 | 8 050 277 | nnxnp | {0-} | nn | np |
|  | **Affx-88862710** | **8 079 267** | **nnxnp** | **{0-}** | **nn** | **np** |
|  | Affx-88862708 | 8 079 762 | nnxnp | {0-} | nn | np |
|  | Affx-88862691 | 8 095 357 | nnxnp | {0-} | nn | np |
| ***LG6A*** | Affx-88886203 | 29 773 273 | hkxhk | {00} | hk | hk |
|  | Affx-88886231 | 29 822 154 | hkxhk | {00} | hk | hk |
|  | **Affx-88886294** | **29 917 230** | **hkxhk** | **{00}** | **hk** | **hk** |
|  | Affx-88886303 | 29 952 036 | hkxhk | {00} | hk | hk |
|  | Affx-88886434 | 30 123 893 | hkxhk | {00} | hk | hk |
| ***LG6B*** | Affx-88816229 | 4 062 319 | hkxhk | {00} | hk | hk |
|  | Affx-88816267 | 4 125 378 | hkxhk | {00} | hk | hk |
|  | **Affx-88816441** | **4 360 876** | **hkxhk** | **{00}** | **hk** | **hk** |
|  | Affx-88877138 | 5 317 721 | hkxhk | {00} | hk | hk |
|  | Affx-88877113 | 5 350 056 | hkxhk | {00} | hk | hk |
| ***LG7A*** | Affx-88878485 | 1 619 115 | hkxhk | {11} | hk | hk |
|  | Affx-88894663 | 1 895 781 | hkxhk | {11} | hk | hk |
|  | **Affx-88894447** | **2 330 648** | **hkxhk** | **{11}** | **hk** | **hk** |
|  | Affx-88894376 | 2 488 286 | hkxhk | {11} | hk | hk |
|  | Affx-88894374 | 2 495 704 | hkxhk | {11} | hk | hk |
