# Supplementary material for: Quantitative trait loci controlling Phytophthora cactorum resistance in the cultivated octoploid strawberry (Fragaria × ananassa)
Source: Hortic Res. 2019 May 1;6:60. doi: 10.1038/s41438-019-0136-4 (PMC6491645; doi:10.1038/s41438-019-0136-4)
Supplement: Supplementary file 7 — Table S5 [file 41438_2019_136_MOESM7_ESM.docx]

**Table S5.** Analysis of variance (ANOVA) revealed no interaction between the three major *Phytophthora cactorum* resistance quantitative trait loci (QTL).

|  | Df | Sum Sq | Mean sq | F value | Pr (>F) | Significance^a^ |
| --- | --- | --- | --- | --- | --- | --- |
| *FaRPc6C* | 1 | 28.92 | 28.919 | 41.955 | 9.34e-10 | *** |
| *FaRPc6D* | 1 | 27.60 | 27.599 | 40.041 | 2.06e-09 | *** |
| *FaRPc7D* | 1 | 27.61 | 27.614 | 40.062 | 2.04e-09 | *** |
| *FaRPc6C:FaRPc6D* | 1 | 0.79 | 0.786 | 1.140 | 0.287 |  |
| *FaRPc6C:FaRPc7D* | 1 | 0.21 | 0.214 | 0.311 | 0.578 |  |
| *FaRPc6D:FaRPc7D* | 1 | 0.27 | 0.271 | 0.393 | 0.531 |  |
| *FaRPc6C:FaRPc6D:FaRPc7D* | 1 | 0.99 | 0.994 | 1.441 | 0.232 |  |
| Residuals | 173 | 119.24 | 0.689 |  |  |  |

^a^ Significance value associated with the marker: ****p*<0.0001
