# Supplementary material for: Quantitative trait loci controlling Phytophthora cactorum resistance in the cultivated octoploid strawberry (Fragaria × ananassa)
Source: Hortic Res. 2019 May 1;6:60. doi: 10.1038/s41438-019-0136-4 (PMC6491645; doi:10.1038/s41438-019-0136-4)
Supplement: Supplementary file 8 — Table S6 [file 41438_2019_136_MOESM8_ESM.docx]

**Table S6.** Analysis of variance (ANOVA) revealed no significant interaction between the response of 15 representative progeny nested within the three sensitivities (lowly diseased, intermediate and highly diseased) and the four *Phytophthora cactorum* isolates tested.

|  | Df | Sum Sq | Mean sq | F value | Pr (>F) | Significance^a^ |
| --- | --- | --- | --- | --- | --- | --- |
| Sensitivity | 1 | 813.5 | 813.5 | 395.269 | <2e-16 | *** |
| Isolate | 3 | 9.5 | 3.2 | 1.537 | 0.2046 |  |
| Sensitivity:Progeny | 13 | 131.5 | 10.1 | 4.914 | 5.97e-08 | *** |
| Sensitivity:Isolate | 3 | 15.4 | 5.1 | 2.500 | 0.0593 |  |
| Sensitivity:Progeny:Isolate | 39 | 103.1 | 2.6 | 1.285 | 0.1252 |  |
| Residuals | 360 | 740.9 | 2.1 |  |  |  |

^a^ Significance value associated with the marker: ****p*<0.0001
